# The COVID-19 Pandemic and Chinese Trade Relations

**DOI:** 10.1007/s11079-022-09692-4

**Published:** 2023-02-03

**Authors:** Jaqueline Hansen, Antonia Kamaliev, Hans-Jörg Schmerer

**Affiliations:** 1 Eberhard Karls University, Tuebingen, Germany; 2 Fern Universität in Hagen, Hagen, Germany; 3 Hausfeld LLP, Duesseldorf, Germany; 4 CESifo, Munich, Germany; 5 IAB, Nuremburg, Germany

**Keywords:** Trade, China, Covid

## Abstract

This paper presents an analysis of the effects of non-pharmaceutical interventions on countries’ bilateral trade with China. Our panel regression results suggest a reduction in monthly Chinese exports to countries that introduced more stringent lockdown measures. We extend our analysis by decomposing observed trade flows into gravity and residual trade components. More stringent lockdowns are associated with less residual trade. Moreover, an event study approach reveals a negative effect of the Covid-19 outbreak in China but this effect vanishes after only 2 months.

## Introduction

Producers and consumers equally benefit from an ample supply of goods in a globalized world but this benefit comes at the cost of higher dependency on foreign markets. Indeed, the recent pandemic unveiled the risks associated with higher international dependency. Plummeting world trade due to the numerous Covid-19 outbreaks disrupted global supply chains and supply of consumer products. Related to this discussion, we study the role of national lockdowns for bilateral trade with China.

We present an econometric analysis of the changes in Chinese trade with particular focus on lockdown policies imposed by China’s trading partners. Lockdowns may have positive and negative effects on bilateral trade. Plummeting demand for Chinese goods can be explained by stricter restrictions on international exchange or less demand by consumers and firms for Chinese products during the lockdown. In contrast, Chinese exports may compensate for declined domestic production caused by lockdown measures as workplace closing or stay at home orders. The net-effect may be negative, positive or even insignificant.

To get a first impression, we conduct a panel regression analysis explaining the difference in monthly exports and imports between 2019 and 2020 by various Covid-19 indicators. The results suggest that countries with stricter lockdown measures imported less goods from China (denoted by Chinese exports). However, we also find the less intuitive result that countries with more stringent lockdowns exported more to China (denoted by Chines imports).

The less intuitive results obtained from the benchmark regression analysis motivate a decomposition analysis of total trade into observed and unobserved trade components. We build this analysis on Brueckner et al. (2020). The authors suggest a standard gravity approach that allows predicting residual trade flows that cannot be explained by the standard gravity determinants. We then confront the residuals with our lockdown measure. This approach allows studying the role of GDP. The residuals are net of expected trade due to the respective country’s level of GDP. Moreover, constructing a counterfactual GDP predicted using the expansion path of GDP before the pandemic. This allows predicting how much of the changes in trade are due to the rapid decline in GDP. The less intuitive result for imports disappears when focusing on residual trade. Lockdowns can be associated with less residual imports and exports.

Another shortcoming in the benchmark analysis is that we are unable to draw a conclusion about the persistence of the lockdown effects. How fast do those effects disappear after launching the lockdown? We shed light on this question using an event study approach that allows distinguishing between short- and long-run effects of the lockdown. This procedures allows us to analyze the effects at different stages of the shock. The launch of the lockdown is associated with an increase in Chinese trade when omitting unobserved heterogeneity. The effects turn negative when controlling for fixed effects in the short-run. These results show that controlling for unobserved heterogeneity matters, which supports the approach proposed by Brueckner et al. (2020).

There are several reasons why we picked China as reference country for our analysis of potential trade effects associated with non-pharmaceutical Covid-19 prevention. China is an important supplier for both consumer products and intermediate goods and most countries in the world somehow rely on trade with China. Moreover, China’s role in the pandemic was special. The country managed bringing the pandemic under control within a rather short period of time by imposing a rigorous zero-Covid strategy while other countries were struggling with less successful lockdown strategies.

**Fig. 1:**
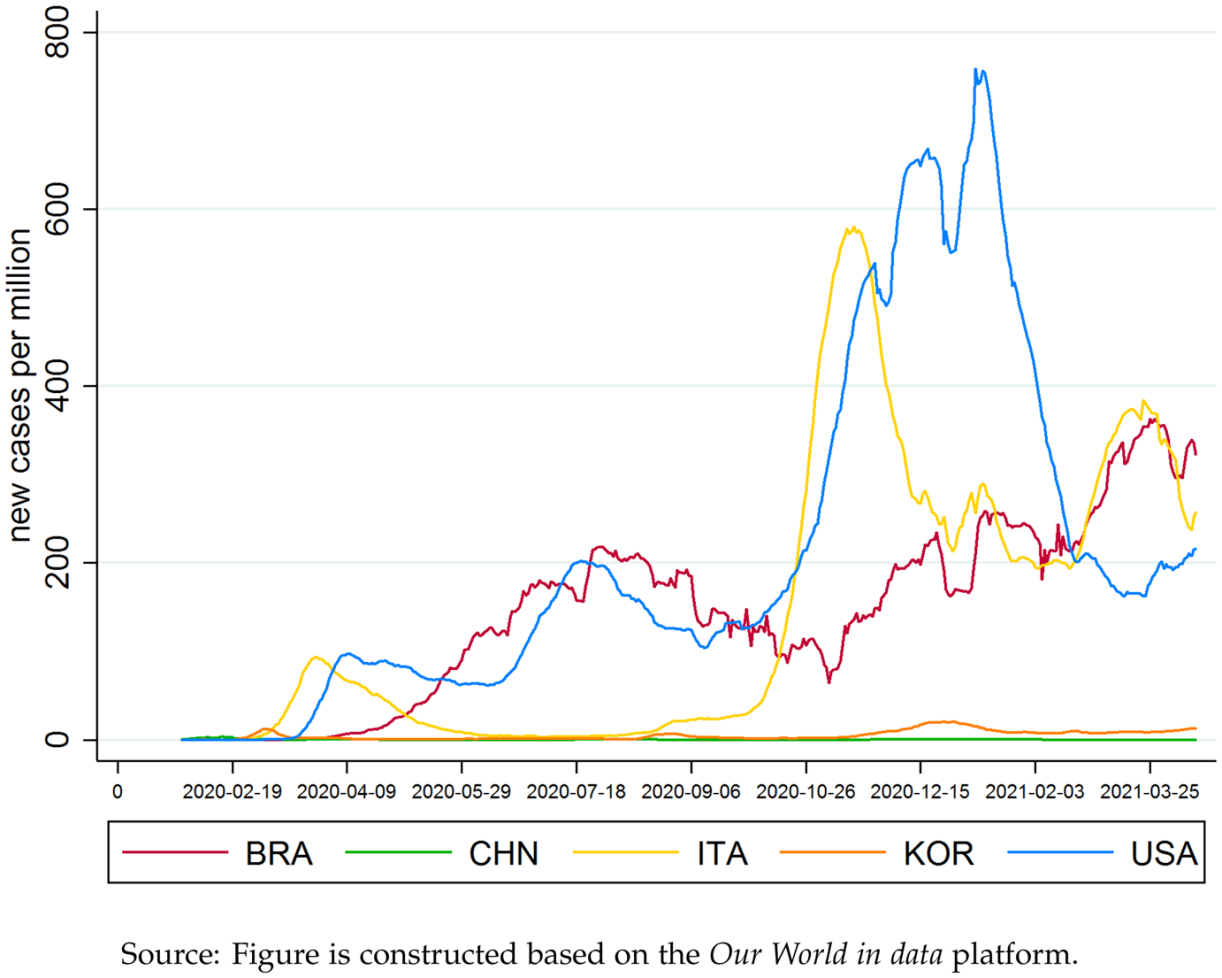
COVID-19 pandemic course for selected countries

Figure 1 compares different transmission rates of Covid-19 between Brazil (BRA), China (CHN), Italy (ITA), South Korea (KOR) and the USA (USA). While China and South Korea reported very low levels of new cases per 1 million residents within one day^1^, countries such as Italy, the USA or Brazil experience very high transmission rates, with sometimes more than 700 new cases per 1 million inhabitants per day.

### Potential Channels Between a Lockdown and Trade with China

China’s reaction to the pandemic was an important predictor for the developments in the rest of the world for various reasons. Firstly, the world became aware of the threat because of China’s rigorous combat against the spread of the virus in early 2020. Secondly, the short-run impact of such an intervention was already observable in China’s GDP growth at the beginning of the crisis. China’s GDP fell by approximately 10 percent in the first quarter of 2020. Thirdly, China is still one of the most important exporters in the world. Thus, the lockdown-effects in China were transmitted through international supply linkages to other countries relying on China. However, the Covid-19 crisis sequentially affected most of China’s trade partners when China was already back to normal, which makes it a good control group.

Our analysis builds upon the hypothesis that lockdowns trigger supply and demand shocks. Companies in countries that introduce a more stringent lockdown may have difficulties producing intermediate and final goods. Some of the domestic production must be substituted by imports from other countries. We argue that China was able to fill the gap by supplying enough goods when production in other countries was plummeting due to a lockdown.

The demand shock operates into the opposite direction. More stringent lockdowns may curb consumers demand through lower wages and less opportunities of consuming commodities. Moreover, the growing sentiments against China may have reinforced this downward trend.

Another potential channel for trade effects due to a lockdown operates through reduced trade costs. Some economies reduced trade barriers for specific goods: Argentina suspended the anti-dumping duties on Chinese medical products. Canada remitted tariffs for specific products if they are imported by health institutions and even the USA exclude a range of medical protective gear and equipment from additional duties.

**Fig. 2:**
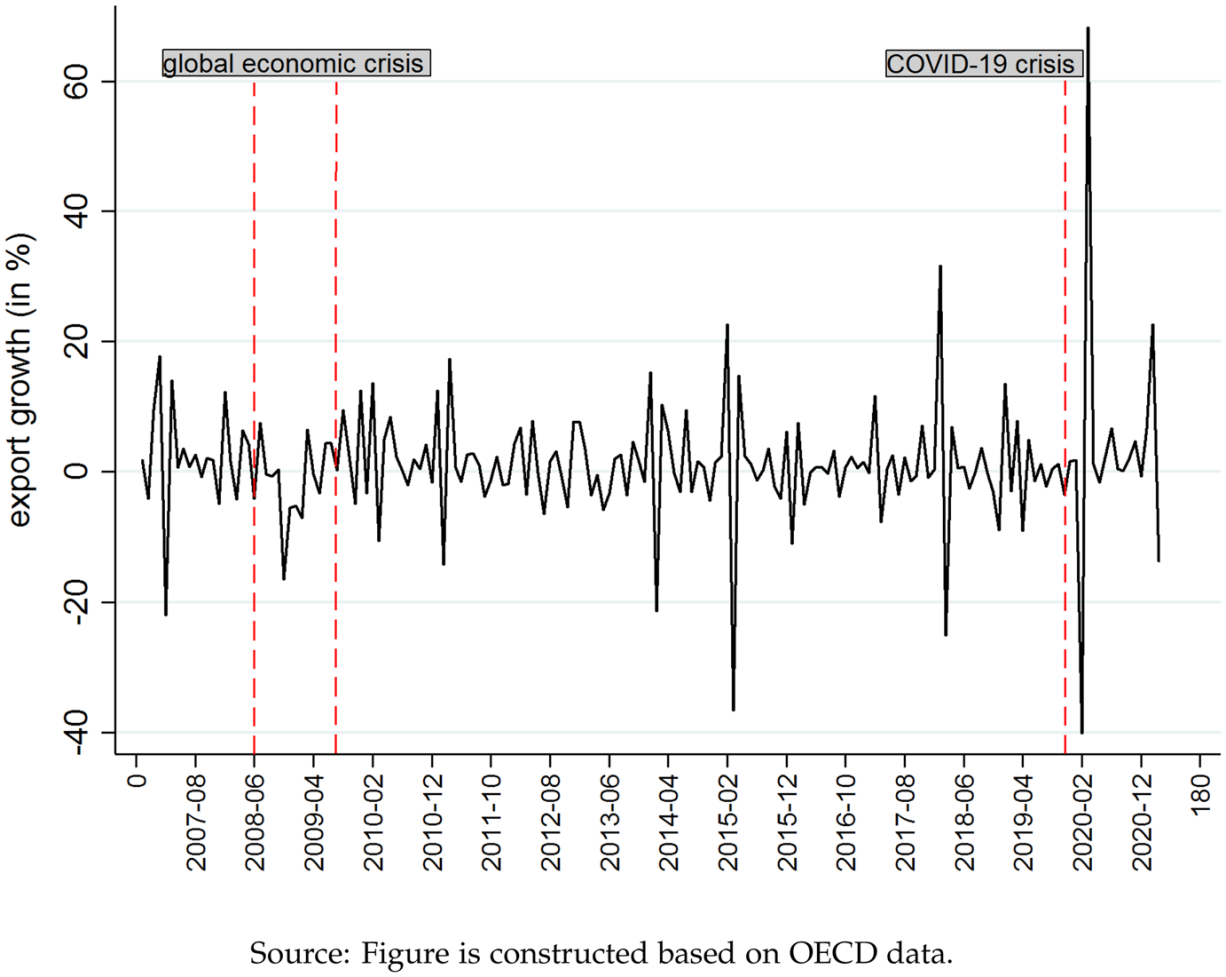
Change in Exports (2006-11 to 2021-2)

Figure 2 gives a first glimpse at the observable effects on bilateral trade with China - China’s monthly export growth between 2006 and beginning 2021.

Compared to the crisis in 2008, the Covid-19 outbreak caused a much more pronounced decline in Chinese export growth of nearly 40%. But by mid-2020, Chinese exports were already back on their initial growth path. The increase in exports more than compensated for the previous decline. The impact of the crisis in 2008 was less pronounced but more persistent.

The analysis is structured as follows: The next subsection provides a literature overview. Subsequently, we present the empirical strategy as well as the data used in our analysis. A discussion of our empirical results is presented in chapter four. The last chapter concludes and give some political implications.

### Related literature

Several studies investigate the trade-off between public health and economic consequences of the Covid-19 pandemic and related lockdown policies in economic models.^2^ Additionally, there is an emerging literature on the affects of the pandemic crisis on international trade. Overall, empirical studies suggest a reduction in the volume of internationally traded goods during the crisis (e.g. Baldwin and Tomiura (2020), Espitia et al. (2021), Gruszczynski (2020), Guan et al. (2020)). Based on a gravity approach, Hayakawa and Mukunoki (2021) identify negative effects on international trade for both importing and exporting countries. Espitia et al. (2021) identify heterogeneous effects across sectors. Industries producing medical products experience positive effects on exports, whereas trade in non-essential durable goods is persistently negative affected. Guan et al. (2020) analyze different lockdown scenarios and their effects on global supply chains. The authors find that trade in intermediaries reacts less sensitive to lockdown restrictiveness than lockdown duration. The overall losses would have been much smaller if lockdown policies were established earlier, stricter and shorter. Verschuur et al. (2021) find stronger effects for economies with strong trading links to China, such as Australia or Malaysia. In contrast, Vietnam seems to benefit from trade diversion effects from China to its own economy. Liu et al. (2021) investigate how the pandemic and lockdown policies in countries affected imports from China. Thus, their paper is closely related to our paper. Overall, the results suggest a dominance of the negative demand effect over the negative supply effect resulting in a reduced volume of imports from China. The effects differ across sectors. Additionally, the authors identify a trade diversion effects to China, if the main trading partner is affected more severely.

We replicate their findings using our own data. However, we depart from their approach by studying the effects on residual trade flows and we are using an event study approach as well. The event study approach shows that the effects are significant in the very short-run and disappear after two months.

## Empirical Strategy

### Panel-data Regression

To get a first impression of the potential relation between lockdown policies and Chinese trade, we regress Covid-19 indicators on Chinese exports and imports. Using monthly panel-data on international trade, we estimate:

1 $$\begin{aligned} \begin{aligned} \Delta trade_{cim} = \alpha&+ \beta _1 log(COVID-19~cases_{im}) + \beta _2 log(COVID-19~deaths_{im}) \\&+ \beta _3 lockdown_{im} + \beta _4 log(population_i) + \beta _{5-7} interaction\\&+ \tau _m [+ \mu _i] +\varepsilon _{im}~~. \end{aligned} \end{aligned}$$

Monthly trade volumes from 2020 are related to the reported values of the same month in 2019. Therefore, the dependent variable $\Delta trade_{cim}$ is defined as the percentage deviation of monthly (*m*) trade flow between China (*c*) and trading partner country *i* from its pre-crisis value observed in the period 2019. Trade flows comprise both imports and exports. Information on the respective country’s lockdown strategy is accounted for by including the *Stringency Index*. This index $lockdown_{im}$ captures various aspects of country-specific interventions against the spread of the virus. Countries reacted differently to the outbreaks. Thus, we include monthly averages of country *i*’s new number of Covid-19 cases $log(COVID-19~cases_{im})$ as additional control for the direct pandemic-impact on an economy. Country specific severity of the pandemic is controlled for by including Covid-19 related deaths $log(COVID-19~deaths_{im})$.

The variable population is included as control for the size of the respective economy. Additionally, we add interactions between country-size and Covid-19 indicators into the regression to see if size matters for the effects of lockdowns on bilateral trade. Seasonal influences are controlled for by including monthly-fixed effects $\tau _{m}$. Unobserved country-specific heterogeneity is controlled for by country-fixed effects, $\mu _i$, in some specifications. The error term is captured by $\varepsilon _{im}$.

### Gravity Approach

We extend the benchmark analysis by disentangling observed trade flows into its predicted and non-predicted trade flows. The method is proposed by Brueckner et al. (2020) who suggest using a gravity approach. The coefficients from the gravity model allow predicting bilateral trade flows in line with the law of gravity. These coefficients are obtained from a regression that fits

2 $$\begin{aligned} \begin{aligned} log(trade)_{cim} = \alpha&+ \beta _1 log(population_{iy}) + \beta _2 log(distance_i)\\&+ \beta _3 log(GDP~p.c._{iy}) + \beta _4 RTA_{ciy}+ \beta _6 border_{ci}\\&+ \lambda _{im} + \varepsilon _{cit}~~. \end{aligned} \end{aligned}$$

The dependent variable $log(trade)_{cim}$ is either the logarithm of trade, the logarithm of imports or the logarithm of exports between China (*c*) and country *i* in month *m*. The dependent variable is explained by the size of trading partner country *i*, approximated by population $log(population_{iy})$ and per capita GDP $log(GDP~ p.c._{iy})$ in year *y*. Trade costs are approximated by the physical distance between China and the respective partner country. A dummy variable for the existence of a Regional Trade Agreement between China and the respective partner country controls for the influence of free trade agreements on bilateral trade. Furthermore, we include a dummy that takes the value one if the respective country has a common border with China $border_{ci}$. The residual $\varepsilon _{cit}$ is the component that cannot be explained by the controls included in the model. Partner-time fixed effects $\lambda _{im}$ address multilateral resistance. The gravity equation is estimated for the years before the crisis, 2017-2019.

The difference between observed bilateral trade flows and the expected trade flow predicted according to the law of gravity is the unexplained residual trade. We expect that the pandemic-effect should mostly be visible in the changes of residual trade. The effects of a lockdown are likely short-run effects with little to no impact on the long-run gravity trade. However, the predicted gravity trade also depends on the two countries’ GDP, which is affected by the crisis. The unexpected decline in GDP is associated with negative changes in gravity trade. To estimate the effect of the pandemic on changes in gravity trade, a counterfactual scenario that represents the predicted situation in 2020 without corona is constructed using counterfactual GDP. Common borders and trade agreements are not affected by the pandemic. In the short-term, population is constant as well. The short-run gravity trade flows are only affected by changes in the decline of GDP caused by the pandemic shock. Compared to the crisis in 2008, the impact of the recent pandemic was much more severe, which should affect the changes in gravity trade but these changes are not necessarily triggered by the lockdowns.

**Fig. 3:**
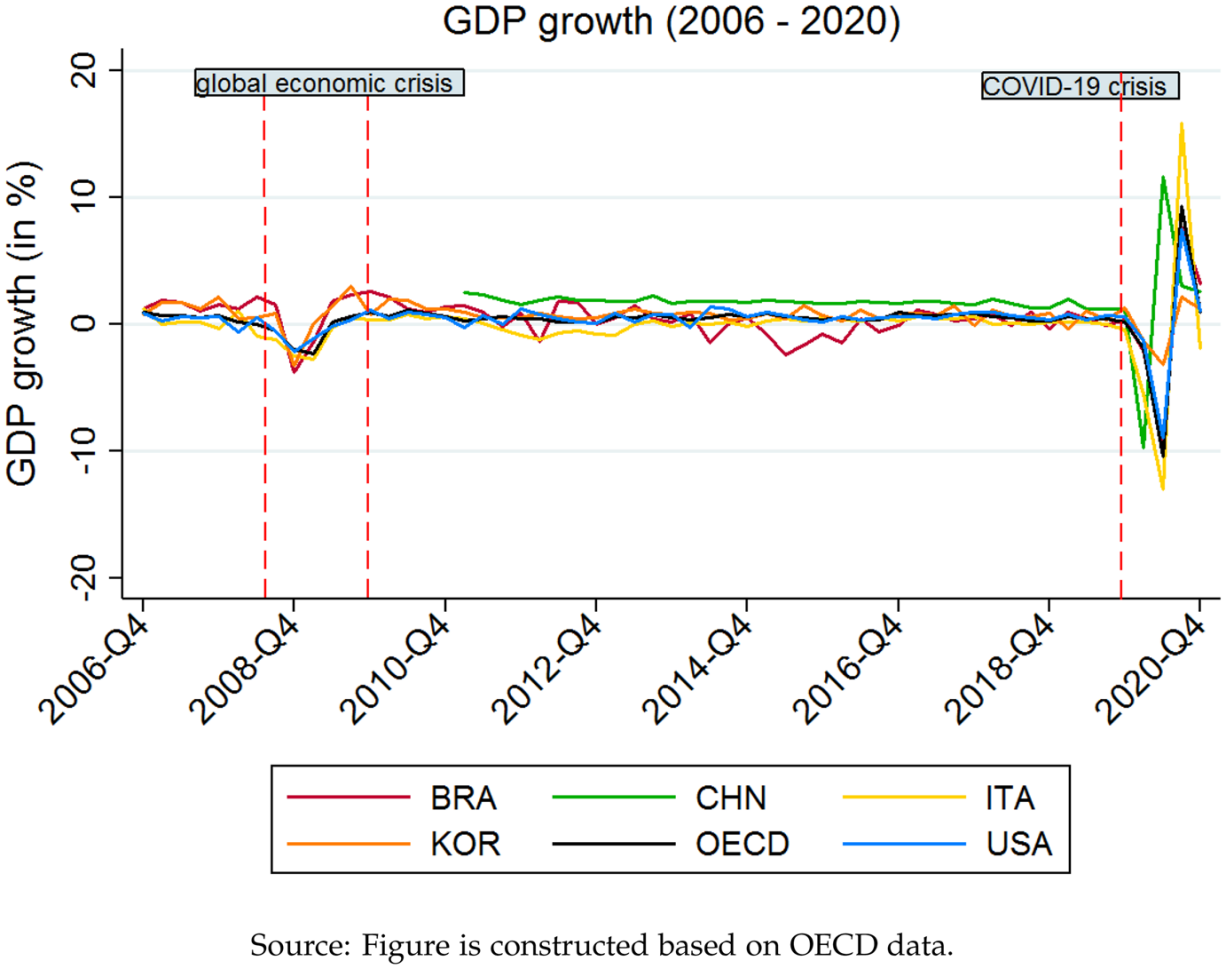
GDP growth (2006 to 2020)

Figure 3 illustrates the strength of the decline in GDP compared to the last global crisis. The decline in GDP growth during the global economic crisis in 2008 was indeed less pronounced compared to the decline observed during the Covid-19 crisis. However, the graphs depicted in Fig. 3 also suggest that the drop was more persistent during the past global economic crisis. After a sharp decrease in 2020, growth rates were recovering relatively fast.

We account for this development by analyzing the role of GDP for the decline in trade. This is done by simulating a scenario that allows predicting counterfactual trade between countries based upon a counterfactual $GDP^{*}$. This counterfactual GDP tries to target a value of GDP reached without the crisis. We are forecasting the evolution of GDP from 2019 onward using a Hodrick-Prescott filter with smoothing parameter set according to the Ravn-Uhlig rule $\xi =1600p^{4}$, where *p* denotes the number of periods within one quarter.^3^ This procedure allows decomposing the time series into trend and a cyclical components.

As a robustness check we forecast the counterfactual non-pandemic $GDP^{*}$ based on a simple linear time-trend prediction. We predict $GDP^{*}$ in each period based upon a common constant, a country-specific intercept and the linear time-trend according to

3 $$\begin{aligned} GDP^{*}_{it}= \gamma _0 + \gamma _1 year + \mu _i~~. \end{aligned}$$

**Fig. 4:**
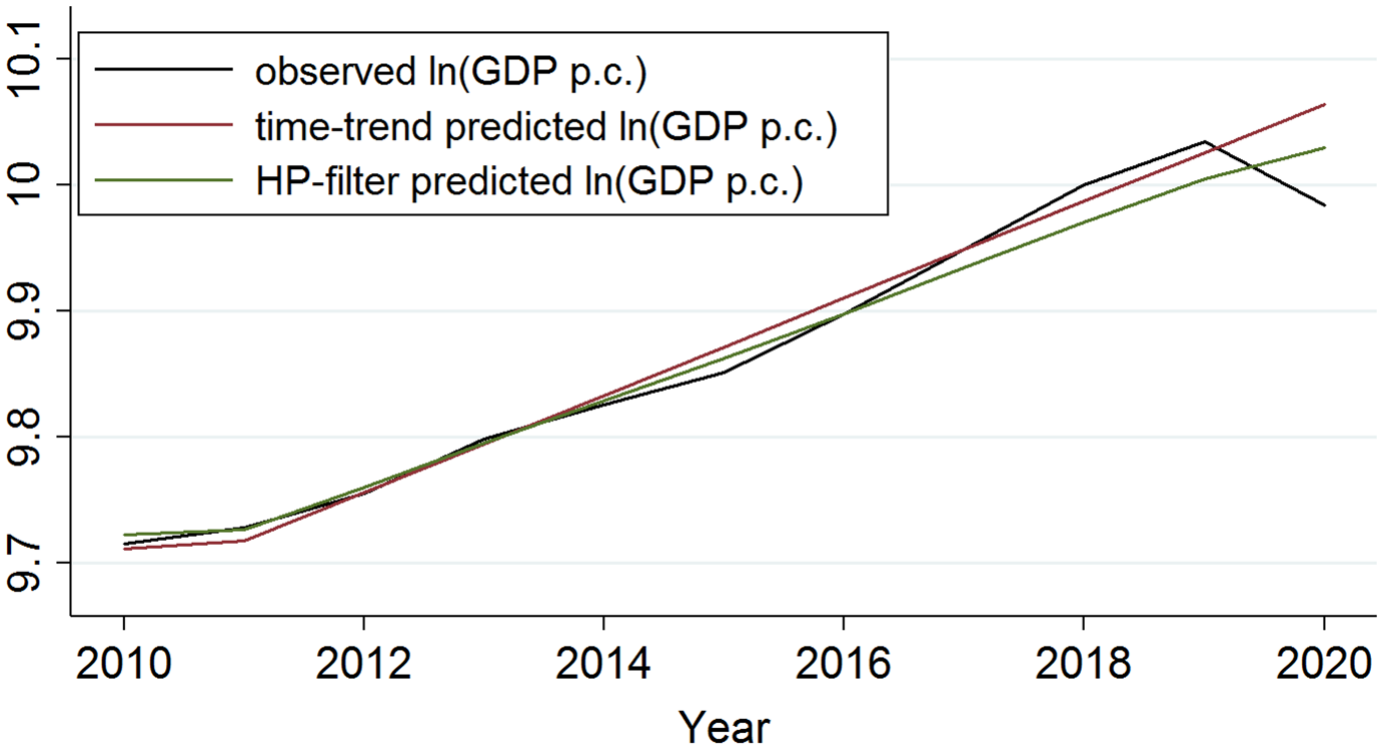
GDP growth (2006 to 2020)

Figure 4 compares observed and counterfactual GDP.

The black line represents cross-country averages of observed GDP between 2010 and 2020 for the whole sample. GDP is declining in 2020. The counterfactual GDP is increasing following the trend in the years before 2020. The green line represents the counterfactual GDP predicted based upon the HP-filter. As expected, the counterfactual GDP is higher in 2020 compared to the observed GDP in the same year.

We estimate gravity Eq. (2) with the observed GDP for the years 2017, 2018 and 2019. Based on these coefficients and the counterfactual GDP values for the respective country, we predict trade volumes for 2020 reflecting world trade without the negative Covid-19 GDP-shock. The predicted values for gravity and residual trade are confronted with the stringency of the respective country’s lockdown. We expect that the trade-effect of the lockdowns is stronger in more stringent countries and that this effect is captured in the residual trade data.

### Event Study

 Long- and short-run effects of the launch of a lockdown in China’s trading partner countries are studied in an event study approach.^4^ This estimation strategy allows analyzing the impact of an unexpected shock in different countries at varying points in time. The advantage of this estimator over the more common diff-in-diff approach is that short- and long-run effects can be distinguished. Moreover, the approach allows accounting for unobserved heterogeneity by including time- and country-fixed effects.

The benchmark event study setup reads

4 $$\begin{aligned} log(trade_{cim}) = \alpha _0 + \sum _{j=2}^{J}\alpha _j(Lag~j)_{im} + \sum _{k=1}^{K} \alpha _k(Lead~k)_{im} + \mu _i + \tau _m + \varepsilon _{im}~~. \end{aligned}$$

Trade with China is explained by various event dummies, fixed-effects and an error term. The event dummies capture the time between the event, which is the first launch of the lockdown in country *i*, and the respective period. The event dummies comprise information on the time till and the time from the event. The leads and lags are defined as follows:

$$\begin{aligned} \begin{aligned} (Lag~J)_{im}&= \mathbb {1}[m\le lockdown_i - J], \\ (Lag~j)_{im}&= \mathbb {1}[m = lockdown_i - j]~ for~ j \in {1, \dots , J-1}, \\ (Lead~k)_{im}&= \mathbb {1}[m = lockdown_i + k]~ for~ k \in {1, \dots , K-1}, \\ (Lead~K)_{im}&= \mathbb {1}[m\ge lockdown_i + K]. \\ \end{aligned} \end{aligned}$$

The dummies *Lag* *J* takes the value one for country *i* when period *m* belongs to one of the periods that is at least *J* periods ahead of the event in this particular country. Suppose that the lockdown in country *i* was launched in August 2020. The *Lag* 1 indicator takes the value 1 in all periods before July 2020. The *Lag* 2 indicator takes the value 1 in all periods before June 2020. The dummies *Lead* 1 replicate the same indicator variable for the period after the event. The dummies *Lag* *j* and *Lead* *k* identify particular dates that are *j* or *k* months before or after the event.

The first lag is defined as the baseline period. Thus, all other coefficients must be interpreted relative to this reference period. Countries that never establish lockdown policies belong to the control group. The dependent variable $log(trade_{cim})$ comprises information about the logarithms of imports, exports or overall trade (imports + exports). All regressions include controls for the time-trend $\tau _m$. In some regressions we consider country fixed-effects $\mu _i$ to control for country-specific unobserved heterogeneity. The error term is denoted by $\varepsilon _{im}$.

## Data

The main Covid-19 indicators are Covid-19 cases and deaths per 1 million inhabitants. The data is taken from Roser et al. (2020) provided by the *Our world in data* platform. Numerous Covid-19 related indicators are included in this data base for 207 countries covering the time from January 2020 to today. However, most economies started to systematically report information only since March 2020. The main pillar of our analysis is a variable that comprises information about the countries’ lockdown policies. The aggregated restrictiveness of monthly lockdown measures in an economy is approximated by the *Stringency Index* provided by Hale et al. (2020b). The index is constructed based upon daily data on various lockdown-categories collected by Hale et al. (2020a) and provided by the *Oxford COVID-19 Government Response Tracker* platform. Eight lockdown variables are included: school closing, workplace closing, cancellation of public events, restriction on gatherings, close public transport, stay at home requirements, restriction on internal movements, and international trade barriers. For more detailed information see Table 6 in the Appendix. All available information on lockdown measures are lumped together and re-scaled to values that range between 0 (no lockdown) and 100 (strictest lockdown).

Using this aggregate stringency index is more convenient than including all different categories by individual dummy variables and there is more variation over time as governments were tightening and loosing the lockdowns step-wise by abolishing different lockdown measures sequentially. The stringency index captures these developments as the higher the stringency index, the higher the number of different lockdown measures imposed by a government.

Finally, data on monthly Chinese imports and exports are taken from the UN Comtrade database that provides numerous indicators related to international trade available at a monthly or annual-level for more than 170 countries covering the time period from 1962 up to today. In our analysis, monthly imports and exports from China to all available trading partners in 2020 and the difference in monthly trade indicators between 2020 and the same month in 2019 are considered.

Daily information on Covid-19 related indicators as well as lockdown policies are transformed from daily into monthly data by taking averages. This procedure neutralizes the impact of outliers in the data and allows us to combine it with the trade data. A summary statistic of the important moments of our data can be found in Table 1.

**Table 1:** Descriptive Statistics

	Obs	Mean	Std. Dev.	Min	Max
Stringency Index	2504	57.568	21.325	0	100
log(COVID-19 cases)	2705	2.151	2.714	-8.740	6.981
log(COVID-19 deaths)	2290	-1.388	2.368	-8.955	3.251
log(export)	807	19.518	2.065	13.595	24.577
export difference to 2019 (in %)	747	0.026	0.335	-0.904	3.744
log(import)	801	18.105	3.316	0	23.430
import difference to 2019 (in %)	738	1.502	18.382	-0.999	450.163
N	2834				

Merging these three data sets allows us to investigate the impact of lockdown policies related to the Covid-19 pandemic on different trade indicators of China with 75 trading partner countries. A list of all countries included in the analysis can be found in Table 7 in the Appendix.

## Results

### Panel-data Regression

First, we present our motivating regression results for the effects of lockdown restrictiveness on trade with China in the respective post-corona months. Trade volume in a month of 2020 is related to the volume in the respective pre-corona month in 2019. Table 2 presents the regression outcomes. In each regression we control for the time trend by including time-fixed effects. The results depicted in columns (3) und (6) additionally include country-fixed effects to control for unobserved heterogeneity at the country-level.

**Table 2:** Motivating regression results

Dependent variable:	*Difference in Chinese exports*			*Difference in Chinese imports*		
	(1)	(2)	(3)	(4)	(5)	(6)
Stringency	-0.329***	-0.771***	-0.306	5.713**	12.286**	4.285
	(0.09)	(0.17)	(0.24)	(2.76)	(5.71)	(10.66)
log(pop)	-0.026***	-0.254***		-0.616**	1.853	
	(0.01)	(0.05)		(0.30)	(1.61)	
log(COVID–19 cases)	-0.005	-0.084***	-0.105***	-0.017	0.567	-0.016
	(0.01)	(0.03)	(0.03)	(0.34)	(0.86)	(0.84)
log(COVID–19 deaths)	0.031**	0.105***	0.109***	-0.412	-1.140	-0.616
	(0.01)	(0.03)	(0.03)	(0.42)	(1.29)	(1.54)
$log(COVID-19~cases) \times log(pop)$		0.031***	0.028***		-0.199	0.024
		(0.01)	(0.01)		(0.23)	(0.25)
$log(COVID-19~deaths) \times log(pop)$		-0.030***	-0.031***		0.262	0.054
		(0.01)	(0.01)		(0.35)	(0.40)
$Stringency \times log(pop)$		0.169***	0.086		-2.542*	-1.001
		(0.05)	(0.07)		(1.40)	(2.68)
constant	0.083	0.861***	-0.043	-1.674	-9.687	-2.194
	(0.13)	(0.22)	(0.14)	(2.23)	(5.88)	(3.67)
Country FE	no	no	yes	no	no	yes
Number of obs.	596	596	596	597	597	597
R within	0.224	0.252	0.277	0.034	0.039	0.022
adj. R	0.203	0.228	0.254	0.008	0.007	-0.008

Standard errors are in parentheses. Significance levels are *p<0.10, **p<0.05, ***p<0.010. The dependent variable is the percentage difference in Chinese export (column (1) - (3)) and imports (column (4) - (6)) to the reference month in 2019. Regressors are logarithmized trading partners’ new COVID-19 cases and deaths per 1 million residents, $log(COVID-19 cases)$ and $log(COVID-19 deaths)$. The variable *Stringency* specifies the restrictiveness of lockdown policies in the trading partners’ economies. Additionally, we control for country-size approximated by population level, *pop*. In each regression we control for the time trend by applying time-fixed effects. In columns (3) and (6) we additionally include country-fixed effects to control for unobserved heterogeneity

The results reported in Table 2 suggest that China’s exports to destination countries that adopted stricter lockdown measures was plummeting, indicated by the highly significant and negative coefficients of *Stringency* presented in columns (1) and (2). These results are robust and can be explained by our theoretical considerations presented in the introduction. A negative effect likely arises due to a negative demand shock. Workers have less income and less opportunities for spending their income when lockdowns are more stringent. Regression (3) includes country fixed-effects as well as the interaction between population and the variables related to Covid-19. Population is country-specific. Thus, the direct effect is absorbed by the fixed-effects. Coefficients are determined by the within-variation of the data. Stringency turns insignificant when controlling for unobserved heterogeneity on the country-level.

The positive coefficient of stringency in columns (4) and (5) suggest that countries with stricter lockdowns tend to export more to China in 2020 compared to the pre-crisis value in 2019. Again a change in the lockdown stringency *within* a country is not significantly associated with more or less imports, as shown by the insignificant coefficient of *Stringency* in (6). One potential explanation for this positive coefficient is the supply shock in countries with lockdowns. Intermediate goods may be exported to China when outsourcing firms at arm’s length are unavailable due to the lockdown.

Chinese exports are also negatively affected by higher levels of new Covid-19 cases in destination countries, shown by the highly significant and negative estimates of $log(COVID-19~cases)$ in columns (2) and (3). These results suggest that destination countries with a higher level of new Covid-19 cases per day import less from China compared to its destination countries with lower levels of new COVID-19 cases (column (2)). Likewise, an increase in the number of new Covid-19 cases within a country reduces import of Chinese goods (column (3)). The direct effect of population size on Chinese exports in 2020 compared to 2019 is also highly significant and negative, hence the drop in export volumes is more pronounced in larger countries. In contrast, Chinese exports tend to be positively affected by the new number of Covid-19 deaths, represented by the significant coefficients of $log(COVID-19~deaths)$ in columns (1) to (3). This effect can be explained by the timing associated with a Covid-19 infection. In case of a severe course, the time span between infection and death is approximately two weeks. Therefore, high levels of infection rates are followed by an increase in death rates about two weeks later. At the same time, governments likely react with strict lockdown measures in a situation where the number of new infections is high, which leads to a reduction in new cases. Imports to China are not significantly affected by the course of the pandemic, as indicated by the insignificant coefficients of Covid-19 cases and deaths in columns (4) to (6).

**Fig. 5:**
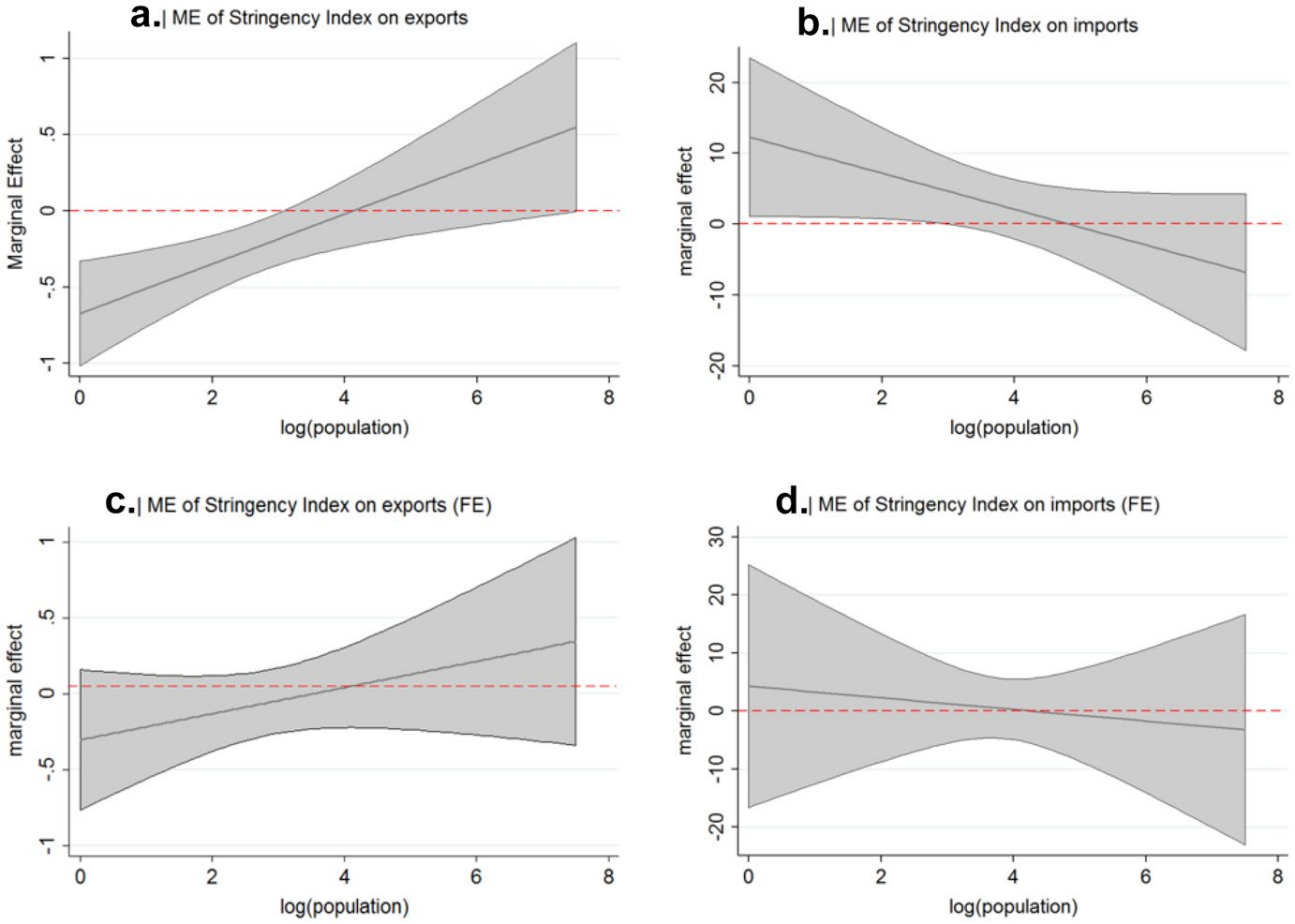
Marginal effects conditional on population size

We are especially interested in the effect of lockdown stringency on trading patterns with China. In the specifications depicted in columns (2), (3), (5) and (6) the overall marginal effect of lockdown stringency must be interpreted conditional on population size. Figure 5 provides a margins plot for lockdown stringency.

Panel a. depicts the marginal effect of lockdown stringency conditional on population size on Chinese exports. This graph allows us to draw conclusions about the effect of lockdown stringency on exports comparing countries with different lockdown policies. Stricter lockdowns in the destination countries are associated with a significant reduction in Chinese exports up to a population size of 99 million inhabitants, which applies to 61 out of 68 countries in our sample. Evaluated at the mean size of a country, an increase in the stringency index by one standard deviation results in a reduction of Chinese exports by around 5.9%. Panel b. gives a graphical representation of the marginal effect of lockdown stringency conditional on population size on imports without controlling for country-specific unobserved heterogeneity. The relation between lockdown stringency and imports is positive up to a population size of around 121 million inhabitants, which applies to 62 out of 68 countries in our sample. However, a change of lockdown stringency within a trading partner is not associated with a significant change in Chinese imports or exports, as depicted in panel c. and d. of Fig. 5.

Up to this point the results suggest that countries establishing stricter lockdown measures tend to import less from China. This result meet our expectations. But the asymmetric result of a positive association of lockdown restrictiveness with Chinese imports is puzzling. To investigate the effect of lockdown policies on trade pattern in more detail, the following section presents the results differentiating between the effect on predicted trade in line with gravity and residual trade.

### Gravity Approach

Table 3 presents the regression results based on monthly data for aggregated trade (columns (1) and (2)), exports (columns (3) and (4)) and imports (columns (5) and (6)). In each regression we control for seasonal trends by including monthly-fixed effects. Multilateral resistance is taken into account by implementing country-month fixed-effects. Moreover, standard errors are clustered at the country-pair level. As a robustness check, the same specification is estimated for three years prior the beginning of the Covid-19 crisis; 2017, 2018 and 2019. For the sake of clarity we stick to estimates for 2017 and 2019 without showing the results for 2018.

**Table 3:** Gravity estimates prior Covid-19 crisis by year (2017 and 2019)

	ln(trade)		ln(exports)		ln(imports)	
	(1)	(2)	(3)	(4)	(5)	(6)
	2017	2019	2017	2019	2017	2019
ln(distance)	-0.328***	-0.441***	-0.173***	-0.208***	-0.881***	-1.021***
	(0.00)	(0.00)	(0.00)	(0.00)	(0.00)	(0.00)
ln(population)	0.965***	0.957***	0.978***	0.954***	1.104***	1.077***
	(0.00)	(0.00)	(0.00)	(0.00)	(0.00)	(0.00)
ln(GDP p.c.)	1.138***	1.146***	1.423***	1.144***	1.070***	1.067***
	(0.00)	(0.00)	(0.00)	(0.00)	(0.00)	(0.00)
Border	-0.695***	-0.790***	-0.041***	-0.593***	-2.440***	-2.382***
	(0.00)	(0.00)	(0.00)	(0.00)	(0.00)	(0.00)
RTA	-0.479***	-0.396***	-0.280***	-0.367***	0.020***	0.320***
	(0.00)	(0.00)	(0.00)	(0.00)	(0.00)	(0.00)
Constant	-4.151***	-3.363***	-9.017***	-5.699***	-0.674***	0.515***
	(0.00)	(0.00)	(0.00)	(0.00)	(0.00)	(0.00)
Number of obs.	787	785	800	798	787	785
R within	0.992	0.995	0.990	0.994	0.957	0.962
adj. R	0.991	0.995	0.989	0.994	0.954	0.958

Standard errors are in parentheses. Significance levels are *p<0.10, **p<0.05, ***p<0.010. Dependent variables are Chinese (imports + exports), *trade*, in columns (1) and (2), Chinese export in columns (3) and (4) and imports in columns (5) and (6). Regressors are logarithmized distance between most populated cities, *ln*(*distance*), logarimized population in million inhabitants, *ln*(*population*), as well as logarithmized GDP p.c. in million USD, *ln*(*GDPp*.*c*.). Furthermore, we control for common border, *Border*, and the presence of regional trade agreements between two trading partners, *RTA*. To control for multilateral resistance we include importer- or exporter-time fixed-effects in each regression. Robust standard errors are clustered at trading-partner level

The estimated coefficients are in line with expectations. The effect of distance on trade is highly significant and negative, suggesting that higher trade cost reduce trade. The negative effect of trade cost, approximated by physical distance between China and trading partner economies, is more pronounced in case of imports than of exports. Country size, approximated by the population level, is associated with more trade, represented by the highly significant and positive coefficient of population. Additionally, this coefficient is close to one in all specifications, which is in line with academic literature.^5^ Similarly, a higher GDP per capita is accompanied by higher trade flows. These size effects are stable across years and the choice of trade indicator. The effect of a common border is negative, which is not in line with intuition. This effect is particularly strong in the case of imports. China imports products mainly from non-neighbouring countries. In contrast, a regional trade agreement magnifies imports to China but the coefficient is significantly negative for overall trade flows and exports. This result may be explainable by the pursued Chinese trade policy: Chinese economic growth is driven by export to a major extend, independently of the existence of RTA between China and their trading partner. In contrast, China imports mainly from countries where trade agreements exist. The test statistics suggest that the estimated specifications explain a high share of observed variation in Chinese trade flows.

Based on the 2019 estimates we predict two different values for aggregated trade flows, exports and imports as well as residuals, respectively. The first prediction rests upon the actual observed GDP in 2020. The second group of trade indicators is predicted based on the counterfactual GDP obtained by applying the HP-filter approach. Table 10 in the Appendix presents the summary statistic for observed and predicted trade, differentiated by the application of observed or counterfactual GDP. In general, predicted trade is higher for counterfactual GDP, driven by the higher GDP values that ignore the negative pandemic effects.

### Lockdown Stringency and Residual Trade

**Fig. 6:**
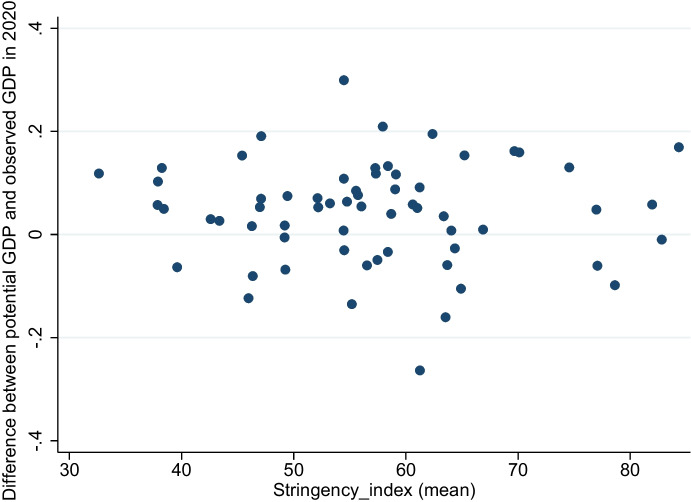
Relationship between average lockdown stringency and difference between observed GDP and its linear prediction

Our gravity approach allows us to investigate whether lockdown measures affect trade flows directly through a decrease in aggregate output or indirectly through channels that are not captured by the gravity equation. In terms of the model, these channels are captured by the “residual trade” that is the difference between observed and predicted trade flows. The observation illustrated in Fig. 6 is that the decrease in output is not systematically associated with lockdown stringency.

This result is rather intuitive as economies that refused imposing a lockdown were not necessarily better off in terms of its economic performance. Thus, there is no obvious reason for an direct effect of lockdowns on gravity trade. This result prompts us to further investigates the effects on non-gravity trade. The decline in GDP may not be systematically related to lockdowns but it affects the residuals obtained from the gravity equation.

To this end, we compute non-gravity - residual trade flows - as the difference between observed monthly trade flows $ln(trade_{m})$ and yearly average trade flow predicted by our gravity estimation $ln(\widehat{trade}_{y})$:

$$\begin{aligned} residual~trade_{m} = ln(trade_{m})- ln(\widehat{trade}_{y}) \end{aligned}$$

This way, we obtain a monthly varying deviation from yearly predicted trade flows that can be related to changes in country-level lockdown policies.

**Table 4:** Correlation between monthly residual trade and lockdown stringency (2020)

	(1)	(2)	(3)
	*residual* *trade*	*residual* *import*	*residual* *export*
stringency	-0.136**	-0.206***	-0.951***
	(0.06)	(0.06)	(0.32)
constant	0.133***	0.158***	0.595***
	(0.03)	(0.04)	(0.19)
Number of obs.	638	645	638
R within	0.275	0.289	0.054
adj. R	0.261	0.276	0.036

Robust standard errors are in parentheses. Significance levels are *p<0.10, **p<0.05, ***p<0.01. Dependent variables is the monthly difference between observed and predicted trade, export and import in 2020. Residuals are regressed on the stringency index, *lockdown* *stringency*. In each regression we control for the time fixed-effects

The results in Table 4 illustrate that the volume of residual trade is negatively associated with lockdown stringency. This reduction in residual trade associated with stricter lockdown measures simultaneously indicate a narrowed gap between effectively observed and potential trade volume. Predicted trade volume, and thereby potential trade related to gravity, is higher than or even exceeds observed trade volume in 2020. However, this finding seems not to be caused by a drop in aggregated output, as shown by the relation presented in Fig. 6. Rather, it shows that the difference in predicted and effectively observed trade is driven by other, unobserved factors. To ensure that these findings are not driven by some unobserved country-specific factors, we also regress the change in residual trade from 2019 to the same month in 2020 on the lockdown stringency indicator.

Table 5 illustrates a very similar pattern in the relative strength of effects. Both specifications reveal that residual trade is negatively associated with a stricter lockdown. This finding confirms our hypothesis that lockdown measures not only affect trade flows through a decrease in aggregate output but also affect the proportion of trade that is not driven by gravity determinants.

**Table 5:** Correlation between monthly residual trade difference (2020-2019) and lockdown stringency

	(1)	(2)	(3)
	residual trade	residual import	residual export
stringency	-0.185***	-0.224***	-0.949**
	(0.06)	(0.07)	(0.39)
constant	0.166***	0.177***	0.563***
	(0.04)	(0.04)	(0.21)
Number of obs.	634	643	634
R within	0.193	0.206	0.033
adj. R	0.177	0.191	0.015

Robust standard errors are in parentheses. Significance levels are *p<0.10, **p<0.05, ***p<0.01. Dependent variables is the monthly change in the difference between observed and predicted trade, export and import between 2019 and 2020. Residuals are regressed on the stringency index, *lockdown* *stringency*. In each regression we control for the time fixed-effects

As an additional robustness check, we conduct an event study comparing specifications ignoring unobserved country-specific heterogeneity exclusively controlling for the time trend and results considering these country-specific effects. Furthermore, this approach allows us to draw a conclusion about the persistence of the effect.

### Event Study

**Fig. 7:**
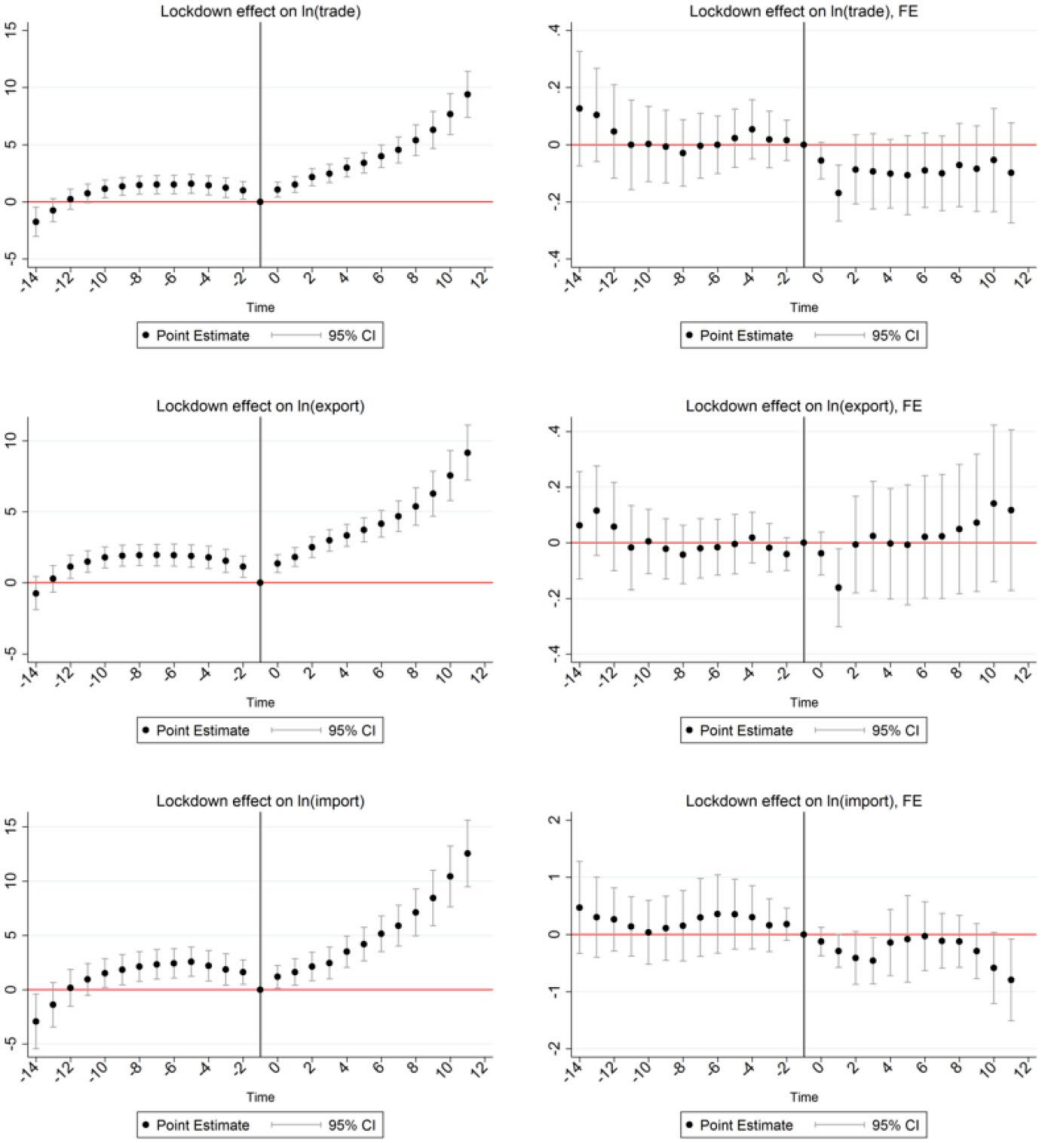
Lockdown on trade, export, import (2019-2020)

In our specification, the event-dummy takes the value one if a country introduces any kind of lockdown, represented by a value of the stringency index > 0. Figure 7 presents the estimation results in an appropriate graph. Each dot represents the coefficient of a specific lead or lag, surrounded by its confidence interval. The black solid line represents the date of the event. Coefficients associated with the months before and after the event must be interpreted relative to this base month 0. Detailed regression result are presented in the Table 8 (Appendix).

Panels on the left are estimated exclusively controlling for the time trend, while panels on the right show estimates including country-fixed effects. The panels in the upper row present the effects on overall trade, the second row focuses on exports and the bottom row on imports.

The coefficient associated with the effect in the first month prior to the lockdown policy is normalized to one and all coefficients must be interpreted relative to this reference period. The significant and positive trade flows in the lockdown period ($time=0$) as well as the in the following periods after the establishment of the lockdown can be interpreted as a trade facilitating effect. This effect holds independently of the choice of the trade measure. The estimations based upon overall trade, exports and imports yield comparable results. In stark contrast to the panel data analysis, countries tend to trade more after imposing a lockdown. However, if we control for country-fixed effects, the effects turn negative: countries imposing any kind of lockdown measure tend to trade less with China shortly after the launch of the lockdown. The effect becomes insignificant two month after the event. Hence, we have identified a short-term negative effect on overall trade flows, exports and imports, but this effect vanishes after only two months. In addition to that, these results allow drawing a second important conclusion: The effect tends to be driven by unobserved heterogeneity among countries.

**Fig. 8:**
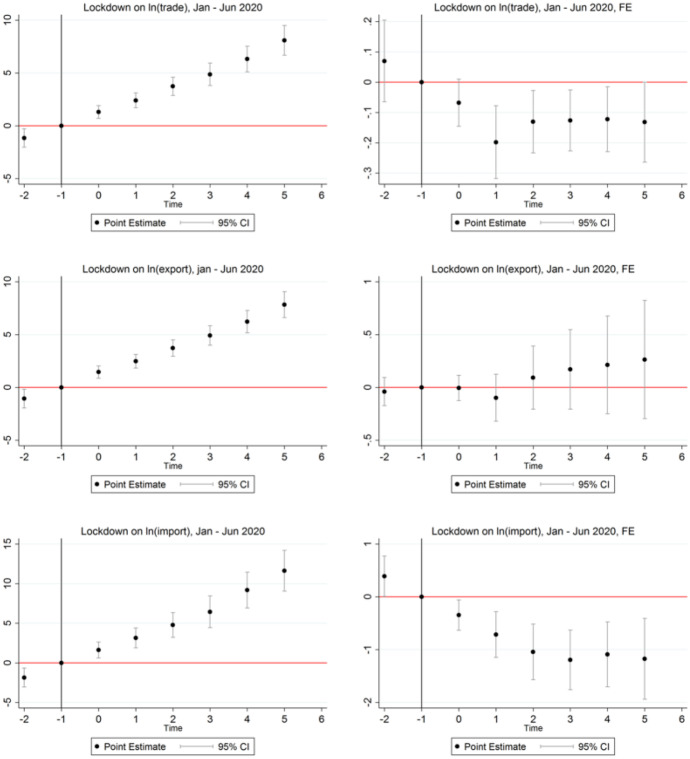
Lockdown on trade, export, import (Jan - Jun 2020)

**Fig. 9:**
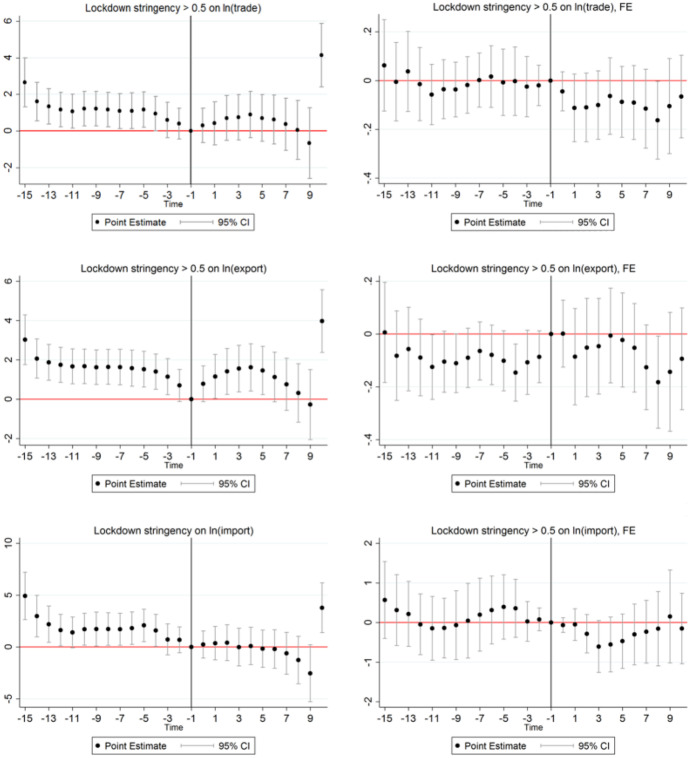
Lockdown stringency > 0.5 on trade, export, import (2019-2020)

**Fig. 10:**
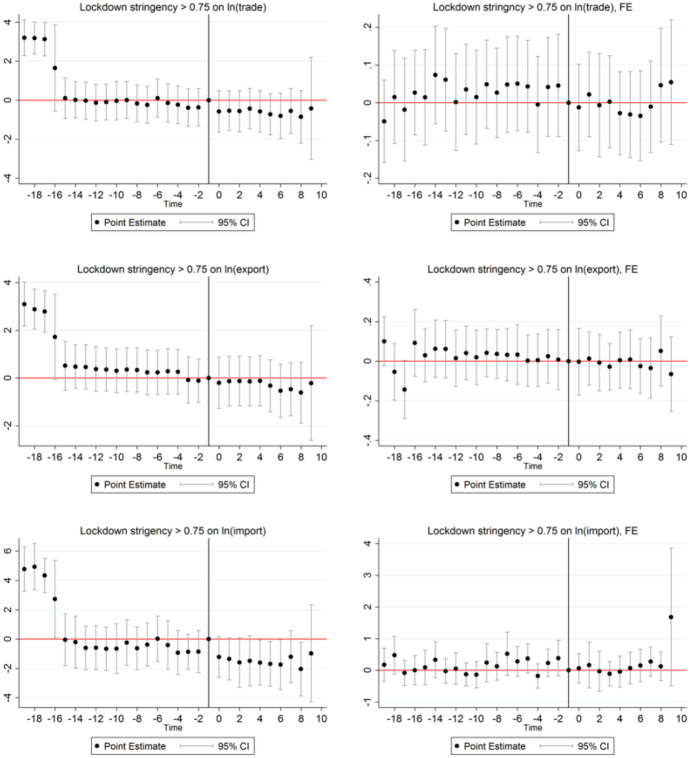
Lockdown stringency > 0.75 on trade, export, import (2019-2020)

We conduct the same analysis for a stricter definition of the event. The threshold for the lockdown event is set to a higher level of 0.50 and 0.75, respectively. The event has a significant effect on trade shortly after the launch but the effect becomes insignificant in the medium- or long-run.^6^ As a further robustness check, we restrict our sample to the first half-year of 2020. The following figure shows the effect of introducing a lockdown in the period January to June 2020. Regression results are presented in Table 9 in the Appendix. Figures 8, 9 and 10 presents results based upon a shorter time frame covering only the month shortly before and after the beginning of the pandemic.

The results based upon this shorter time-frame are slightly different. Neglecting unobserved heterogeneity results in a positive effect of lockdowns as represented by the highly significant coefficients in the panels on the left. In contrast, controlling for country-fixed effects (panels at the right), total trade flows as well as imports are negatively affected by the event.

The effects on exports become insignificant in the shorter sample. Nevertheless, the overall pattern remains unchanged: the positive effect of lockdown on trade seems to be mainly driven by unobserved heterogeneity among countries. These estimates support the results rest upon the gravity approach: changes in trade due to lockdown policies seem to be mainly driven by unobserved heterogeneity. The negative effect of stricter lockdown measures on trade volumes become observable in a reduction of residual trade.

## Conclusion

In this paper, we use recent estimation techniques from the empirical trade literature to study the impact of the Covid-19 pandemic on Chinese trade flows. Using panel-data estimators, we find a significant negative relationship between stricter lockdown measures in export destination countries and Chinese exports. To further investigate the driving factors behind the change in trade flows, we implement a gravity estimation that allows predicting Chinese trade flows. The advantage of this approach is its ability to differentiate observed trade flows into trade predicted by the gravity forces and the so-called residual trade. To account for the decline in macroeconomic output caused by the pandemic, we built a counterfactual GDP that extrapolatesthe trend before the pandemic. We find a robust negative relationship between lockdown stringency in the destination countries and the yearly change in residual trade. Stricter lockdowns seem to reduce the difference between observed and gravity based predicted trade flows, indicating a trade potential higher than effectively observed trade in pandemic periods. This finding is novel, it highlights that the pandemic affected trade flows not only through changes in aggregate output but also through the portion of bilateral trade that is not explained by country observables. To asses the overall impact of the initial lockdown as well as persistence of the effect, we implement an event-study for the years 2019 and 2020. This approach allows us to normalize the timing of the pandemic for all countries. When controlling for unobserved effects at the time- and country-level, our results show an initial economic downturn and no significant changes in the long run.

Overall, the effect is significant but small as it disappears after a rather short period of time. One should keep in mind that the lockdowns studied in this paper were one-sided lockdowns as China managed to keep the virus under control with only a few exceptions. It is likely that the impact on aggregate trade would have been much more severe with more stringent lockdowns in China during the period of analysis in our paper.

## Data Information and Robustness

**Table 6:** Lockdown categories & definition

Category	Category name	Category description and coding
$C_1$	*school closing*	$C_1=0$: no measures
		$C_1=1$: recommend closing, or all school open with alterations resulting in significant differences compared to usual.
		$C_1=2$: require closing (some levels or categories)
		$C_1=3$: require closing all levels
$C_2$	*workplace closing*	$C_2=0$: no measures
		$C_2=1$: require closing (or work from home)
		$C_2=2$: require closing (or work from home) for some sectors or worker categories
		$C_2=3$: require closing (or work from home) all-but-essential workplaces
$C_3$	*cancel public events*	$C_3=0$: no measures
		$C_3=1$: recommend cancelling
		$C_3=2$: require cancelling
$C_4$	*restrictions on gatherings*	$C_4=0$: no measures
		$C_4=1$: restrictions on gatherings above 1000 people
		$C_4=2$: restrictions on gatherings between 101 - 1000 people
		$C_4=3$: restrictions on gatherings between 11 - 100 people
		$C_4=4$: restrictions on gatherings of 10 people or less.
$C_5$	*close public transport*	$C_5=0$: no measures
		$C_5=1$: recommend closing (or significantly reduce volume)
		$C_5=2$: require closing (or prohibit most citizens from using it)
$C_6$	*stay at home requirements*	$C_6=0$: no measures
		$C_6=1$: recommend not leaving home
		$C_6=2$: require not leaving home with exceptions for daily exercise, grocery shopping, and ’essential’ trips
		$C_6=3$: require not leaving home with minimal exceptions
$C_7$	*restrictions on internal movement*	$C_7=0$: no measures
		$C_7=1$: recommend not to travel between regions/cities
		$C_7=2$: internal movement restrictions in place
$C_8$	*International travel controls*	$C_8=0$: no measures
		$C_8=1$: Screening
		$C_8=2$: Quarantine arrivals from high-risk regions
		$C_8=3$: Ban on arrivals from some regions
		$C_8=4$: Ban on all regions or total border closure
$H_1$	*Public information campaigns*	$H_1=0$: No COVID-19 public information campaign
		$H_1=1$: public officials urging caution about COVID-19
		$H_1=2$: coordinated public information campaign (e.g. across traditional and social media)

**Table 7:** List of Countries

Nr.	Country Name	Nr.	Country Name
1	Australia	37	Japan
2	Azerbaijan	38	Kenya
3	Barbados	39	Kyrgyz Republic
4	Belarus	40	Latvia
5	Belgium	41	Lesotho
6	Belize	42	Lithuania
7	Bolivia	43	Luxembourg
8	Bosnia and Herzegovina	44	Mauritius
9	Botswana	45	Mexico
10	Brazil	46	Moldova
11	Bulgaria	47	Myanmar
12	Canada	48	Namibia
13	Colombia	49	Netherlands
14	Congo	50	New Zealand
15	Costa Rica	51	Norway
16	Croatia	52	Pakistan
17	Cyprus	53	Paraguay
18	Czech Republic	54	Peru
19	Denmark	55	Philippines
20	Ecuador	56	Poland
21	Egypt	57	Portugal
22	El Salvador	58	Romania
23	Estonia	59	Rwanda
24	Finland	60	Senegal
25	Gambia	61	Serbia
26	Georgia	62	Slovak Republic
27	Germany	63	Slovenia
28	Greece	64	South Africa
29	Guatemala	65	Spain
30	Guyana	66	Sweden
31	Hungary	67	Switzerland
32	Iceland	68	Turkey
33	India	69	Uganda
34	Ireland	70	Ukraine
35	Israel	71	United Kingdom
36	Italy	72	United States
		73	Uruguay
		74	Zambia
		75	Zimbabwe

### Event Study

**Table 8:** Lockdown (stringency $>$ 0) on trade, exports, imports

	(1)	(2)	(3)	(4)	(5)	(6)
	In(trade)	In(trade)	In(export)	In(export)	In(import)	In(import)
lead14	-1.751***	0.127	-0.726	0.062	-2.917**	0.471
	(0.65)	(0.10)	(0.59)	(0.10)	(1.28)	(0.40)
lead13	-0.748	0.104	0.282	0.115	-1.389	0.303
	(0.52)	(0.08)	(0.47)	(0.08)	(1.05)	(0.35)
lead12	0.228	0.047	1.126***	0.058	0.161	0.263
	(0.45)	(0.08)	(0.42)	(0.08)	(0.86)	(0.28)
lead11	0.740*	-0.001	1.494***	-0.018	0.945	0.140
	(0.41)	(0.08)	(0.39)	(0.08)	(0.74)	(0.26)
lead10	1.135***	0.002	1.783***	0.004	1.524**	0.037
	(0.40)	(0.07)	(0.38)	(0.06)	(0.68)	(0.28)
lead9	1.341***	-0.006	1.905***	-0.022	1.833**	0.109
	(0.40)	(0.06)	(0.38)	(0.05)	(0.71)	(0.28)
lead8	1.461***	-0.029	1.946***	-0.043	2.124***	0.153
	(0.40)	(0.06)	(0.38)	(0.05)	(0.70)	(0.31)
lead7	1.506***	-0.004	1.954***	-0.021	2.336***	0.298
	(0.41)	(0.06)	(0.39)	(0.05)	(0.69)	(0.34)
lead6	1.505***	-0.001	1.944***	-0.017	2.422***	0.358
	(0.42)	(0.05)	(0.40)	(0.05)	(0.69)	(0.35)
lead5	1.579***	0.023	1.884***	-0.005	2.571***	0.354
	(0.42)	(0.05)	(0.40)	(0.05)	(0.69)	(0.31)
lead4	1.438***	0.054	1.792***	0.018	2.211***	0.300
	(0.43)	(0.05)	(0.40)	(0.05)	(0.72)	(0.28)
lead3	1.232***	0.018	1.538***	-0.018	1.868**	0.163
	(0.44)	(0.05)	(0.42)	(0.04)	(0.74)	(0.23)
lead2	0.994**	0.015	1.131***	-0.041	1.612***	0.180
	(0.39)	(0.04)	(0.39)	(0.03)	(0.58)	(0.14)
lag0	1.060***	-0.056*	1.349***	-0.039	1.194**	-0.125
	(0.33)	(0.03)	(0.32)	(0.04)	(0.53)	(0.12)
lag1	1.507***	-0.169***	1.808***	-0.162**	1.625***	-0.290**
	(0.36)	(0.05)	(0.34)	(0.07)	(0.62)	(0.14)
lag2	2.162***	-0.087	2.502***	-0.007	2.134***	-0.414*
	(0.39)	(0.06)	(0.37)	(0.09)	(0.67)	(0.23)
lag3	2.480***	-0.093	2.986***	0.024	2.457***	-0.461**
	(0.41)	(0.07)	(0.39)	(0.10)	(0.74)	(0.20)
lag4	2.981***	-0.101*	3.340***	-0.004	3.497***	-0.141
	(0.41)	(0.06)	(0.40)	(0.10)	(0.73)	(0.29)
lag5	3.396***	-0.107	3.725***	-0.008	4.193***	-0.081
	(0.45)	(0.07)	(0.43)	(0.11)	(0.79)	(0.38)
lag6	3.981***	-0.090	4.161***	0.021	5.148***	-0.029
	(0.50)	(0.07)	(0.48)	(0.11)	(0.84)	(0.30)
lag7	4.538***	-0.101	4.694***	0.023	5.901***	-0.112
	(0.58)	(0.07)	(0.55)	(0.11)	(0.96)	(0.24)
lag8	5.390***	-0.071	5.375***	0.049	7.113***	-0.122
	(0.69)	(0.07)	(0.67)	(0.12)	(1.10)	(0.23)
lag9	6.290***	-0.084	6.277***	0.072	8.436***	-0.291
	(0.83)	(0.08)	(0.81)	(0.12)	(1.30)	(0.24)
lag10	7.676***	-0.054	7.562***	0.141	10.434***	-0.587*
	(0.92)	(0.09)	(0.90)	(0.14)	(1.43)	(0.31)
lag11	9.407***	-0.099	9.167***	0.117	12.538***	-0.797**
	(1.02)	(0.09)	(0.99)	(0.15)	(1.56)	(0.36)
constant	7.237***	6.318***	5.939***	5.950***	5.662***	3.818***
	(0.56)	(0.07)	(0.50)	(0.08)	(1.07)	(0.32)
Country FE		yes		yes		yes
Number of obs.	1,634	1,634	1,677	1,677	1,634	1,634
R within	0.143	0.273	0.175	0.281	0.102	0.053
adj. R	0.117	0.251	0.151	0.259	0.075	0.024

**p* < 0.10; ***p* < 0.05; ****p* < 0.010

**Table 9:** Lockdown (stringency $>$ 0) on trade, exports, imports (Jan - Jun 2020)

	(1)	(2)	(3)	(4)	(5)	(6)
	In(trade)	In(trade)	In(export)	In(export)	In(import)	In(import)
lead2	-1.154***	0.070	-1.061**	-0.039	-1.859***	0.391**
	(0.44)	(0.07)	(0.45)	(0.07)	(0.61)	(0.19)
lag0	1.307***	-0.068*	1.458***	-0.005	1.626***	-0.346**
	(0.31)	(0.04)	(0.30)	(0.06)	(0.51)	(0.14)
lag1	2.412***	-0.198***	2.480***	-0.098	3.149***	-0.713***
	(0.36)	(0.06)	(0.33)	(0.11)	(0.64)	(0.22)
lag2	3.739***	-0.130**	3.731***	0.093	4.784***	-1.042***
	(0.44)	(0.05)	(0.39)	(0.15)	(0.80)	(0.26)
lag3	4.873***	-0.126**	4.927***	0.171	6.447***	-1.193***
	(0.54)	(0.05)	(0.47)	(0.19)	(1.02)	(0.28)
lag4	6.327***	-0.122**	6.230***	0.214	9.191***	-1.089***
	(0.62)	(0.05)	(0.54)	(0.23)	(1.15)	(0.31)
lag5	8.098***	-0.132*	7.837***	0.264	11.627***	-1.174***
	(0.72)	(0.07)	(0.62)	(0.28)	(1.31)	(0.38)
constant	6.830***	6.531***	6.273***	6.099***	5.333***	4.548***
	(0.31)	(0.02)	(0.32)	(0.03)	(0.37)	(0.07)
Country FE		yes		yes		yes
Number of obs.	388	388	402	402	388	388
R within	0.348	0.309	0.372	0.337	0.266	0.067
adj. R	0.328	0.287	0.353	0.317	0.243	0.037

**p* < 0.10; ***p* < 0.05; ****p* < 0.010

### Descriptives Predicted Trade, Exports and Imports

**Table 10:** Descriptive Statistics - predicted trade for 2020 based on 2019 data

	Obs	Mean	Std. Dev.	Min	Max
**Observed Trade**					
*ln*(*trade*)	752	6.499	2.068	0.434	11.033
*ln*(*export*)	772	6.035	2.065	-0.220	10.761
*ln*(*import*)	752	4.597	3.299	-13.816	10.367
**Predicted Trade based on observed GDP**					
*predicted* *ln*(*trade*)	748	6.278	2.109	1.085	10.748
*predicted* *ln*(*export*)	748	5.959	1.986	0.941	10.54
*predicted* *ln*(*import*)	748	4.216	3.471	-8.658	9.285
*residual* *ln*(*trade*)	728	0.063	0.260	-1.337	1.379
*residual* *ln*(*export*)	748	0.047	0.283	-1.371	1.483
*residual* *ln*(*import*)	728	0.093	0.965	-6.364	8.621
**Predicted Trade based on counterfactual GDP**					
$predicted~ ln(trade_c)$	748	6.326	2.118	1.397	10.811
$predicted~ ln(export_c)$	748	6.007	1.994	1.253	10.605
$predicted~ ln(import_c)$	748	4.261	3.464	-8.339	9.411
$residual~ ln(trade_c)$	728	0.021	0.303	-1.680	1.360
$residual~ ln(export_c)$	748	0.000	0.326	-1.714	1.464
$residual~ ln(import_c)$	728	0.053	0.967	-6.503	8.302
**Differences in residuals between 2019 and 2020**					
*observed GDP*					
$diff.~trade~residuals_{2019-2020}$	722	-0.067	0.262	-1.493	1.442
$diff.~export~residuals_{2019-2020}$	744	-0.048	0.287	-1.644	1.442
$diff.~import~residuals_{2019-2020}$	722	-0.085	1.034	-9.699	8.516
*counterfactual GDP*					
$diff.~trade~residuals_{2019-2020, c}$	722	0.025	0.303	-1.785	1.474
$diff.~export~residuals_{2019-2020, c}$	744	-0.001	0.329	-1.625	1.784
$diff.~import~residuals_{2019-2020, c}$	722	-0.046	1.038	-9.380	8.655
$N$	772				

## Footnotes

1 The numbers never exceed 4 or 20 new cases per 1 million inhabitants.

2 See for example Atkeson (2020), Alvarez et al. (2020), Eichenbaum et al. (2020), Farboodi et al. (2020), Krueger et al. (2020).

3 We are thankful to one critical referee mentioning a more recent paper by Hamilton (2018). The author criticizes the HP-filter approach and suggests using an alternative approach based upon regressing current outcomes on lagged outcomes. However, we stick to the more common approach as the difference between the outcomes based upon the HP-filter and the method proposed by Hamilton (2018) are almost similar in our application.

4 We implement the event study approach following Clarke and Schythe (2020).

5 See for example Head et al. (2010) and Head and Mayer (2014).

6 Results are available upon request.
